# Complete Genome Sequences of the Novel Cluster BP Phages Infecting Streptomyces sanglieri, AxeJC, Cumberbatch, Eastland, Eklok, HFrancette, Ignacio, Piccadilly, and Vondra

**DOI:** 10.1128/mra.00751-22

**Published:** 2022-09-14

**Authors:** Sreemoye Nath, Ahmad Sulaiman, Jindanuch Maneekul, Swapan Bhuiyan, Sonya Layton, Carolina Menchaca, Subhayu Nayek, Juan Acosta, Alejandro Coronado, Alexandra Drake, Isaac Eastland, Monserrat Gallegos, Misti Gutierrez-Langa, Naomi Jefferson, Kennede Johnson, Erin Klokker, Marie Muniz, Diana Hernandez Olmos, Grace Pascarella, Tucker Richardson, Taylor Setliff, Alyssa N. Stiles, Eliza Sun, Melisa Tokel, Jessica M. Vondra, Lee E. Hughes

**Affiliations:** a Department of Biological Sciences, University of North Texas, Denton, Texas, USA; Queens College CUNY

## Abstract

The Streptomyces sanglieri bacteriophages AxeJC, Cumberbatch, Eastland, Eklok, HFrancette, Ignacio, Piccadilly, and Vondra form a novel actinobacteriophage cluster, BP. These siphoviruses have circularly permuted genomes with an average size of 37,700 bp and a GC content of 71%. Each genome contains approximately 58 protein-coding genes, with no tRNAs.

## ANNOUNCEMENT

More than 300 *Streptomyces* phage genomes have been sequenced and grouped, based on gene content similarity (GCS), into 18 phage clusters, BA to BR (https://phagesdb.org). Here, we announce eight genomes that form a novel cluster, BP. Phages AxeJC, Cumberbatch, Eastland, Eklok, HFrancette, Ignacio, Piccadilly, and Vondra were isolated in 2019 and 2020 from different topsoil samples collected around Texas (Table 1).

**TABLE 1:** Streptomyces sanglieri cluster BP phage genome information

Phage name	Sampling location	Genome length (bp)	GC content (%)	Coverage (no. of sequencing reads)	No. of genes	GenBank accession no.	SRA accession no.
AxeJC	Denton, TX (33.212222N, 97.149444W)	37,765	71.4	181	58	ON392164	SRX14443482
Cumberbatch	Lewisville, TX (33.021862N, 97.02347W)	37,566	71.3	1,422	59	MT451982	SRX14443494
Eastland	Rowlett, TX (32.8881N, 96.5752W)	37,536	71.2	134	58	ON392165	SRX14443498
Eklok	Denton, TX (33.211259N, 97.14836W)	37,409	71.2	6,105	58	MT521991	SRX14443499
HFrancette	Denton, TX (33.211551N, 97.145429W)	38,234	71.2	167	59	ON456341	SRX14443512
Ignacio	El Paso, TX (31.784983N, 106.448778W)	38,137	71.1	2,401	58	MT451980	SRX14483210
Piccadilly	Denton, TX (33.211917N, 97.149361W)	37,544	71.2	1,685	58	OL455886	SRX14483238
Vondra	Denton, TX (33.212232N, 97.1485W)	37,129	71.2	1,550	57	MT451981	SRX14485103

**FIG 1:**
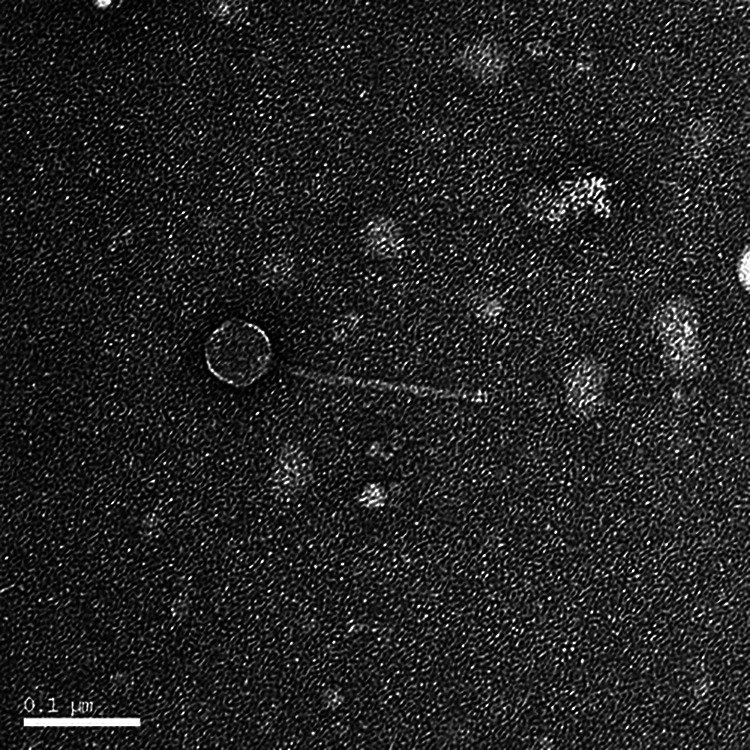
Electron micrograph of *Streptomyces* phage Eastland, an example cluster BP phage with *Siphoviridae* morphology. A high-titer lysate placed on Formvar-coated grids was negatively stained with UranyLess electron microscopy stain (Electron Microscopy Sciences). Imaging was performed with a Phillips EM420 microscope using a conventional tungsten filament operated at 120 kV.

These eight phages were isolated using Streptomyces sanglieri UNT16F27A and standard procedures (1). Briefly, nutrient broth inoculated with Streptomyces sanglieri was added to soil samples and allowed to incubate at 30°C for 2 days before the mixture was centrifuged (4,000 × *g*), the supernatant was filtered (0.22-μm filter), and the filtrate was spread plated with Streptomyces sanglieri on nutrient agar supplemented with 10 mM MgCl_2_, 8 mM Ca(NO_3_)_2_, and 0.5% glucose. Plaques were purified by three rounds of plating, resulting in uniform circular plaques that ranged from 0.5 to 1.5 mm in diameter. Negative-staining transmission electron microscopy revealed all phages to be siphoviruses, with a capsid diameter and a tail length of approximately 60 and 190 nm, respectively (Fig. 1).

DNA was extracted using the Promega Wizard DNA cleanup system. Sequencing libraries were constructed using the NEBNext Ultra II DNA library preparation kit and sequenced using an Illumina MiSeq system (v3 reagents), generating 150-base single-end reads. All reads were assembled into single contigs as described by Russell (2) using Newbler v2.9 (3) and Consed v29 (4). Sequencing results and genome characteristics are listed in Table 1. All isolates contain circularly permuted genome ends, as determined by even distribution of reads across the assembly, with an average length of about 37,700 bp and GC contents above 71%. Based on shared GCS exceeding 85%, as assessed using the GCS tool at the Actinobacteriophage Database (https://phagesdb.org), these phages are grouped together to form a new actinobacteriophage phage cluster, BP (5, 6).

All bioinformatic tools were used with default parameters. Initial autoprediction of coding regions was performed using GeneMark v3.25 (7) and GLIMMER v3.02b (8). Subsequently, annotations were curated manually using DNA Master v5.23.6 (9), Phamerator (10), BLAST (11), Starterator v1.0.1 and v1.2 (http://phages.wustl.edu/starterator), and PECAAN (https://blog.kbrinsgd.org/about-us/). No tRNA genes were identified by ARAGORN v1.2.41 (12) and tRNAscan-SE v2.0 (13). Functions for each coding sequence were evaluated using NCBI BLASTp v2.9 (11), HHpred v3.2 (14), Phamerator (10), and PECAAN. Membrane proteins were predicted using TMHMM v2.0 (15). The genomes contain about 58 genes each, with 19 to 22 genes for which a function could be identified.

The genes are highly conserved in all eight phages, although Vondra contains two small clusters of genes for which there are no homologues in the Actinobacteriophage Database. Each phage contains a predicted serine integrase gene, which suggests that these phages are temperate. The integrase of cluster BP phages was most similar to those of phages in cluster BC. The putative endolysin genes are highly conserved in all eight phages (89 to 100% protein similarity) and share similarity with endolysins found in members of 10 other *Streptomyces* phage clusters.

### Data availability.

The GenBank and SRA accession numbers are listed in Table 1.
